# Artificial intelligence adoption and organizational efficiency: the mediating role of organizational learning capability and moderating role of digital literacy

**DOI:** 10.3389/frai.2026.1891713

**Published:** 2026-09-30

**Authors:** Hayford Asare Obeng, Richard Arhinful, Leviticus Mensah, Abdullah Sarwar

**Affiliations:** 1 Department of Business Administration, Onbes Kasim Kibris University, Nicosia, Cyprus; 2 Department of Business Administration, World Peace University, Nicosia, Cyprus; 3 Center of Accounting, Finance and Economics, Multimedia University, Cyberjaya, Selangor, Malaysia; 4 Centre for Management and Marketing Innovation (CMMI), COE for Business Innovation and Communication, Faculty of Management, Multimedia University, Cyberjaya, Malaysia

**Keywords:** artificial intelligence, digital literacy, organizational efficiency, organizational learning capability, Resource-Based View (RBV)

## Abstract

**Introduction:**

Strategic and organizational solutions are becoming essential due to the global demand for digital transformation, sustained operational efficiency, and the rapid advancement of artificial intelligence (AI) technologies. This study examines the influence of artificial intelligence adoption on organizational efficiency, with digital literacy as a boundary condition and organizational learning capability serving as a mediator.

**Methods:**

The study utilized a quantitative cross-sectional survey-based design. A multistage sampling technique was used to collect data from employees working in the Ghanaian banking sector. Data collected from 388 employees from the banks were utilized in an empirical study employing structural equation modeling. Both direct and indirect relationships were assessed.

**Results:**

The findings revealed that the level of artificial intelligence adoption has a significant and positive influence on both organizational efficiency and organizational learning capability. The results further showed that organizational learning capability has a significant and positive influence on organizational efficiency and partially mediates the relationship between artificial intelligence adoption and organizational efficiency. Additionally, the results revealed that digital literacy moderates the relationships between artificial intelligence adoption and organizational efficiency, as well as the relationships between organizational learning capability and organizational efficiency.

**Conclusion:**

This study introduces a novel theoretical framework that connects the hitherto overlooked linkages between artificial intelligence adoption, organizational learning capability, digital literacy, and organizational efficiency within the banking sector of an emerging economy. By integrating these constructs into a unified model, the study clarifies the mechanisms and boundary conditions through which AI adoption translates into organizational efficiency, thereby extending the Resource-Based View in an emerging-market context.

## 1 Introduction

With capabilities that improve decision-making, customer service, fraud detection, and operational efficiency, artificial intelligence (AI) is becoming a key component of competitive strategy in the banking industry (Benlian et al., 2022; Enholm et al., 2022). A recent McKinsey Global Survey (McKinsey, 2023) indicates that firms identified as high performers in AI have implemented both traditional and generative AI across more business areas than their counterparts—especially in service delivery, product creation, and risk management. These trends highlight AI’s transformative potential to improve business processes. Nonetheless, alongside these benefits, considerable concerns remain, including job displacement, algorithmic bias, and the inability of some organizations to fully exploit AI because of contextual limitations such as skill gaps and infrastructure barriers (Ntoutsi et al., 2020; Cheng et al., 2022). Recent empirical research similarly shows that AI improves organizational efficiency through advances in process automation, predictive analytics, decision-making quality, and operational agility (Obeng et al., 2026a,b). Despite this, the advantages of AI are not uniformly distributed among organizations, as effective implementation relies on supplementary organizational capabilities, employee skills, and technological preparedness rather than mere technology adoption (Dwivedi et al., 2021; Kalyani and Gupta, 2023; Eskandarany, 2024). These contrasting findings indicate that AI adoption should be analyzed alongside the organizational factors that enable companies to convert technology expenditures into quantifiable results.

In emerging nations like Ghana, the banking industry has rapidly embraced digitalization in recent years, propelled by regulatory changes and consumer demands. Financial institutions are increasingly adopting AI-powered solutions for tasks such as customer verification, credit assessment, and digital customer engagement (Kaluarachchi and Sedera, 2024). Despite these initiatives, a critical gap remains: few empirical studies examine how AI adoption produces concrete organizational outcomes—especially efficiency—within African banking institutions. Most research is conducted in technologically advanced economies, limiting the applicability of existing findings (Zhang et al., 2021; Gigerenzer, 2022). Moreover, extant research has predominantly focused on consumer adoption of AI-driven banking services, fintech advancements, and digital evolution. At the same time, relatively little attention has been given to how AI improves internal organizational efficiency through organizational capabilities. This limitation is especially evident in emerging nations, where institutional limitations, digital infrastructure, and workforce preparedness vary significantly from those in developed economies (Dwivedi et al., 2021; Kalyani and Gupta, 2023). Thus, empirical evidence from Ghana offers a valuable opportunity to extend understanding of AI-driven organizational transformation in a relatively underexplored context.

The existing literature has gaps in clarifying the mechanisms and boundary conditions that determine AI’s impact on organizational performance. To address this, we present Organizational learning capability as a mediating variable. Organizational learning capability refers to an organization’s ability to acquire, share, and apply knowledge effectively for strategic advantage (Jerez-Gomez et al., 2005; Begum et al., 2025). It plays a pivotal role in helping organizations internalize AI’s advantages by converting technology resources into practical insights and process innovation. While earlier research has indicated favorable correlations between AI adoption and organizational performance, significantly less attention has been devoted to explaining the organizational mechanisms by which AI generates efficiency gains. According to the Resource-Based View, organizations with stronger learning capabilities are better able to transform AI-generated insights into continuous improvement, innovation, and operational efficiency.

Additionally, we propose digital literacy as a moderating variable, defined as the skills, understanding, and dispositions needed to use digital tools effectively in the workplace (Ng, 2012; Mardiana and Yakub, 2025). We argue that higher levels of digital literacy strengthen the relationship between AI adoption and organizational learning capability, enabling employees to interact with AI systems more confidently and facilitating learning processes within the business. Similarly, digitally literate employees are better able to interpret AI-generated insights, adapt to AI-enabled work processes, and maximize the organizational value derived from AI investments, making digital literacy an important boundary condition in AI implementation (Benlian and Pinski, 2025).

This study is grounded in the Resource-Based View (Barney, 1991), which asserts that a firm’s performance depends on its ability to utilize distinctive, valuable, and non-replicable resources. In this context, AI adoption is a technological resource, but its value depends on intangible resources such as learning capability and workforce digital proficiency (Arhinful et al., 2026a). Thus, the Resource-Based View provides a suitable theoretical lens for explaining why organizations using comparable AI technology may show varying levels of efficiency based on organizational capacities and employee competencies.

By positioning the study within Ghana’s banking industry, we respond to recent calls for AI research in non-Western, underrepresented contexts (Guercini, 2023; Ivanov, 2023). Ghana offers a compelling case because of its relatively advanced fintech landscape in sub-Saharan Africa, alongside persistent challenges in digital capacity and knowledge diffusion. This duality makes it an ideal context to investigate the bright and dark aspects of AI adoption. This study examines the relationship between AI adoption and organizational efficiency by exploring the mediating role of organizational learning capability and the moderating effect of digital literacy in Ghana’s banking sector. The research aims to explain not only whether AI adoption enhances organizational efficiency but also the mechanisms and conditions under which it does. Thus, it attempts to answer the following research questions:

*RQ1:* What is the relationship between organizational AI adoption and organizational efficiency?

*RQ2:* Does organizational learning capability mediate the relationship between organizational AI adoption and organizational efficiency?

*RQ3:* Does digital literacy moderate the relationship between organizational AI adoption and organizational efficiency?

*RQ4:* Does digital literacy moderate the relationship between organizational learning capability and organizational efficiency?

The research offers four significant contributions to the existing literature. First, it extends the growing AI literature by providing empirical evidence from Ghana’s banking industry, a context often overlooked in AI and organizational management studies. Second, it advances understanding of how AI adoption enhances organizational efficiency by establishing the mediating role of organizational learning capability. Third, it recognizes digital literacy as an essential boundary condition that strengthens AI-driven organizational efficiency. Ultimately, by integrating AI adoption, organizational learning capability, and digital literacy into the Resource-Based View, the research enhances the understanding of how technological and organizational resources jointly foster organizational efficiency in emerging countries.

## 2 Theoretical background and hypothesis development

### 2.1 Theoretical foundation

The Resource-Based View provides a robust theoretical framework for examining the strategic significance of AI in influencing organizational performance. The Resource-Based View posits that firms that possess and effectively use valuable, rare, inimitable, and non-substitutable (VRIN) resources can achieve sustainable competitive advantage (Varadarajan, 2023; Arhinful et al., 2026b). In AI, the technology is not inherently advantageous; instead, its strategic value depends on an organization’s ability to integrate it into a distinctive configuration of competencies and human knowledge (Olan et al., 2022; Jarrahi et al., 2023).

This study indicates that employing AI is a strategic technological advantage. Nevertheless, consistent with the Resource-Based View, the mere existence of AI capabilities does not guarantee enhanced organizational performance (Neiroukh et al., 2024; Obeng et al., 2026a,b). The significance lies in integrating, assimilating, and applying these technologies, which primarily rely on organizational learning mechanisms and employee competency.

This necessitates careful consideration of organizational learning capability as an essential intermediary. This discussion focuses on organizational learning capability, defined as an organization’s ability to gather, comprehend, and apply information for adaptive and innovative purposes (Jerez-Gomez et al., 2005). From a Resource-Based View perspective, OCL is an intangible asset that enables firms to transform technological inputs, such as AI, into operational and enhanced processes (Jarrahi et al., 2023), thereby increasing efficiency.

Moreover, digital literacy significantly affects the effectiveness of AI applications. It is a form of human capital, an additional intangible asset emphasized in the Resource-Based View that enables individuals to interact effectively with digital systems (Ng, 2012). Enhanced digital literacy augments employees’ absorptive capacity, hence facilitating the adoption of AI and the exploration of its potential applications through learning. AI and digital literacy engage both technical and strategic dimensions to influence resource allocation within organizations (Jankovic and Curovic, 2023).

This study uses the Resource-Based View to conceptualize AI as a strategic resource whose effectiveness depends on internal capabilities and is moderated by contextual competencies, rather than as a singular solution. This lens addresses calls in contemporary literature to reconsider digital transformation from a resource-centric viewpoint rather than solely a technological one (Benlian et al., 2022; Rai et al., 2019). Resource-Based View provides a thorough theoretical framework for examining the twin influences of AI, its potential for operational improvement, and the risks of inefficiency due to insufficient supporting mechanisms such as organizational learning capability and digital literacy.

### 2.2 Hypotheses development

#### 2.2.1 AI adoption, organizational learning capability and organizational efficiency

AI has emerged as a transformative tool for enhancing organizational efficiency in domains that require speed, accuracy, and scalability. AI technologies are widely used in the banking sector to strengthen operations and improve efficiency by automating repetitive processes, enhancing client engagement, detecting fraud, and generating predictive insights (McKinsey, 2023; Syam and Sharma, 2018). AI’s ability to analyze large datasets and perform repetitive tasks autonomously may lead to substantial cost savings and improved productivity (Huang and Rust, 2021).

The Resource-Based View posits that technology must be demonstrated as a valued and scarce organizational asset to enhance performance (Barney, 1991). AI can serve as a valuable instrument if its implementation yields discernible improvements in operational outcomes. Empirical research, such as Neiroukh et al. (2024) and Badghish and Soomro (2024), shows that adopting AI correlates positively with organizational efficiency. Businesses that integrated AI into their core processes reported enhanced operational agility, improved process quality, and expedited service delivery (Wang et al., 2022). Ghasemaghaei (2019) observed that data-driven AI systems significantly enhance decision-making precision, reduce task redundancy, and markedly improve resource efficiency across several sectors. In turn, despite AI’s strong potential to enhance business performance, its implementation largely depends on internal competencies and contextual enablers.

However, the link between AI adoption and efficiency is not always linear or universally positive. AI may introduce complexities such as system misalignment, employee resistance, and dependence on opaque algorithmic processes (Benlian et al., 2022; Gigerenzer, 2022). Contextual constraints may limit AI’s efficiency in emerging economies such as Ghana, where digital infrastructure and organizational preparedness can vary, leading to inefficiencies or operational disruptions.

The practical implementation of AI in organizations extends beyond mere automation; it requires an environment that fosters the simultaneous growth of the organization and the technology, promoting learning and adaptation (Chowdhury et al., 2024). In this context, organizational learning capability, defined as the organization’s ability to acquire, disseminate, and utilize knowledge, holds significant importance (Jerez-Gomez et al., 2005). The effective application of AI technology generates new data streams, feedback systems, and knowledge repositories that strengthen a company’s learning culture and flexibility (Han et al., 2024; Grundner and Neuhofer, 2021).

According to the Resource-Based View paradigm, an organization’s learning capability is a valuable, intangible asset that enables firms to derive strategic advantages from technological acquisitions (DiBella et al., 1996; Lei, 1997; Tippins and Sohi, 2003). Integrating AI deployment with a robust learning orientation promotes ongoing enhancement, knowledge codification, and innovation within corporate processes (Rana et al., 2022). Empirically, Jöhnk et al. (2021) discovered that organizations integrating AI into their learning systems exhibited greater adaptability and responsiveness to market changes. Duan et al. (2019) assert that AI systems can foster a culture of data-driven learning, enabling staff to utilize insights from intelligent systems to refine policies and optimize procedures.

This relationship is not guaranteed. Because of inadequate knowledge-sharing practices and a detrimental learning environment, AI may create isolated domains of expertise, hindering comprehensive organizational learning (Zhang et al., 2021). Moreover, if employees cannot evaluate the insights generated by AI systems, the organization may fail to use the knowledge effectively, limiting its transformative potential.

Consequently, while implementing AI holds significant potential to enhance organizational learning capabilities, the effectiveness of this integration depends on the company’s absorptive capacity and digital proficiency to convert AI inputs into valuable insights. Based on the above discussions, the following hypotheses were formulated:

*H1:* AI adoption is significantly and positively associated with organizational efficiency.

*H2:* AI adoption is significantly and positively associated with organizational learning capability.

#### 2.2.2 Organizational learning capability and organizational efficiency

An organization’s ability to generate, collect, distribute, and combine data to improve performance is called its organizational learning capability (Jerez-Gomez et al., 2005). In the modern business landscape, particularly in the banking industry, organizational learning capability has become a strategic necessity because rapid technological advancements, evolving customer demands, and legislative changes require ongoing adaptation. Rather than merely acquiring knowledge, the emphasis is on integrating learning into organizational processes and culture to promote continuous responsiveness and adaptation (Hung et al., 2010; Giannakos et al., 2022).

Broadly defined as an institution’s capacity to provide high-quality services and outcomes while minimizing waste and resource use (Delaney and Huselid, 1996), organizational efficiency is strongly influenced by an organization’s ability to learn from internal and external sources. When organizational learning capacity improves, it leads to better information sharing, encouragement of experimentation, stronger problem-solving, and less duplicated work (Goh, 2003). These results immediately help improve services, cost control, and operations—essential qualities of efficiency in sectors such as banking.

Studies have found empirical support for the influence of learning capability on efficiency. For instance, Marchiori et al. (2022) and Li et al. (2022) reported that companies with stronger learning capacities were better able to use technology assets to enhance operational performance. Gomes et al. (2022) found that organizational learning improves internal processes and service delivery, increasing efficiency. Using a multi-sector sample, Alegre and Chiva (2008) found a significant positive correlation between organizational learning capacity and performance, especially when institutionalized learning was strategically supported and embedded throughout the company.

In many different situations, the linkage remains erratic and non-automatic. The extent to which an organization’s learning capability yields efficiency depends on environmental dynamics, organizational scale, and leadership style. In banking, antiquated legacy systems or strict hierarchical structures can impede the transformation of knowledge into efficiency unless organizations implement intentional structural and cultural changes (Pacheco-Cubillos et al., 2024; Lyons et al., 2007). Therefore, although the literature confirms the fundamental relevance of organizational learning capability in improving efficiency, its practical expression depends on the effective application of learning within the company. From the discussions above, the following hypothesis was developed:

*H3:* Organizational learning capability is significantly and positively associated with organizational efficiency.

#### 2.2.3 Mediating role of organizational learning capability

The adoption of AI holds significant promise to enhance organizational efficiency (Olan et al., 2022); however, realizing these benefits typically depends on a corporation’s internal learning systems. Initially dynamic and complex, AI systems require organizational adaptation, learning, and technological integration to align their capabilities with operational objectives (Rai et al., 2019; Jöhnk et al., 2021). Organizational learning capability is a crucial mediator in this context, enabling investments in AI to translate into higher output.

Organizations with robust learning capabilities are more inclined to assimilate and integrate ideas generated by AI systems (Sun and Anderson, 2010). These firms are better equipped to understand AI outputs, make data-driven decisions, and adjust procedures based on real-time feedback. Organizational learning capability serves as a conduit that enhances the integration of AI technologies into a business’s daily operations (Jerez-Gomez et al., 2005; Jarrahi et al., 2023). This learning system enables firms to optimize procedures, eliminate redundancy, and enhance accuracy, essential attributes of organizational effectiveness.

Empirical research substantiates this mediating pathway. According to Duan et al. (2019), AI technologies improve corporate performance only when integrated with strong absorptive and learning capacity. Organizational learning, as articulated by Aboelmaged and Hashem (2019), mediates the relationship between digital innovation and performance outcomes in highly competitive businesses. These findings suggest that without an appropriate learning framework, organizations may underutilize the transformative potential of AI or even be harmed by it.

Moreover, organizations with high organizational learning capacity are better able to identify deficiencies in AI use, implement corrective measures, and disseminate successful AI applications across multiple divisions (Olan et al., 2022). This iterative approach, grounded in organizational learning, ensures that AI adoption is an ongoing process rather than a one-time event, adapting to company requirements (Jarrahi et al., 2023; Wijnhoven, 2022). Organizations lacking learning ability may find that AI technology conflicts with practical realities, resulting in inefficiencies or unsuccessful initiatives.

Organizational learning capability plays a mediating role that is both strategic and instrumental, as it shapes the extent to which AI use delivers expected efficiency gains. Organizations can optimize and sustain the benefits of AI integration by fostering a culture that prioritizes learning, experimentation, and continuous development. From the ongoing discussions, we developed the following hypothesis:

*H4:* Organizational learning capability mediates the relationship between AI adoption and organizational efficiency.

#### 2.2.4 Moderating role of digital literacy

Digital literacy is a key contextual factor that strongly influences how much AI enhances organizational efficiency. AI systems require end users to utilize the tools and integrate their results into established procedures (Pimenov et al., 2023). Consequently, digital literacy enables employees to fully leverage the benefits of AI; without it, underutilization or resistance may ensue, limiting efficiency improvements.

Organizations with higher levels of digital literacy are more likely to achieve operational efficiency with AI implementation, as employees can effectively engage in data-informed decision-making and innovation. Duan et al. (2019) argue that technologically proficient staff support proactive adaptation and AI use; however, Celik (2023) emphasizes that insufficient digital literacy can hinder effective AI implementation, resulting in technical inefficiency. Nguyen and Malik (2022) observed that digital skills strengthen AI’s impact on operational outcomes and service quality. In contrast, Rana et al. (2022) found that insufficient digital capabilities negate the anticipated performance benefits of AI investments. The results affirm that digital literacy shapes the relationship between AI adoption and efficiency, thereby enhancing or constraining outcomes depending on its presence.

Similarly, digital literacy connects organizational efficacy with learning capacity. Organizational learning capability facilitates knowledge acquisition and transformation (Imran et al., 2016); however, its effectiveness depends on the workforce’s ability to navigate digital environments (Noonan et al., 2017). Organizational learning capability activities, which encompass knowledge sharing and experiential learning, are executed more efficiently and are more likely to improve organizational processes in environments characterized by high digital literacy.

Conversely, inadequate digital literacy may limit learning opportunities and affect operational outcomes. Knowledge management systems or technology-based learning tools may pose challenges for staff members. Valk and Planojevic (2021) emphasized that effective participation in knowledge-sharing events relies on digital fluency. In contrast, López-Nicolás and Meroño-Cerdán (2011) noted that digital competency fosters a learning-oriented culture and enhances efficiency outcomes (Aboobaker and Ka, 2021).

Digital literacy influences organizations’ effectiveness in implementing AI and using online learning communities, serving two concurrent moderating functions. By investing in digital literacy, banks undergoing ongoing digital transformation and data-driven operations can fully optimize the efficiencies provided by AI and machine learning capabilities. From the ongoing discussions, we developed the following hypotheses:

*H5:* Digital literacy moderates the relationship between AI adoption and organizational efficiency.

*H6:* Digital literacy moderates the relationship between organizational learning capacity and organizational efficiency.

The study model below was built using the literature review and hypothesis development (Figure 1).

**Figure 1:**
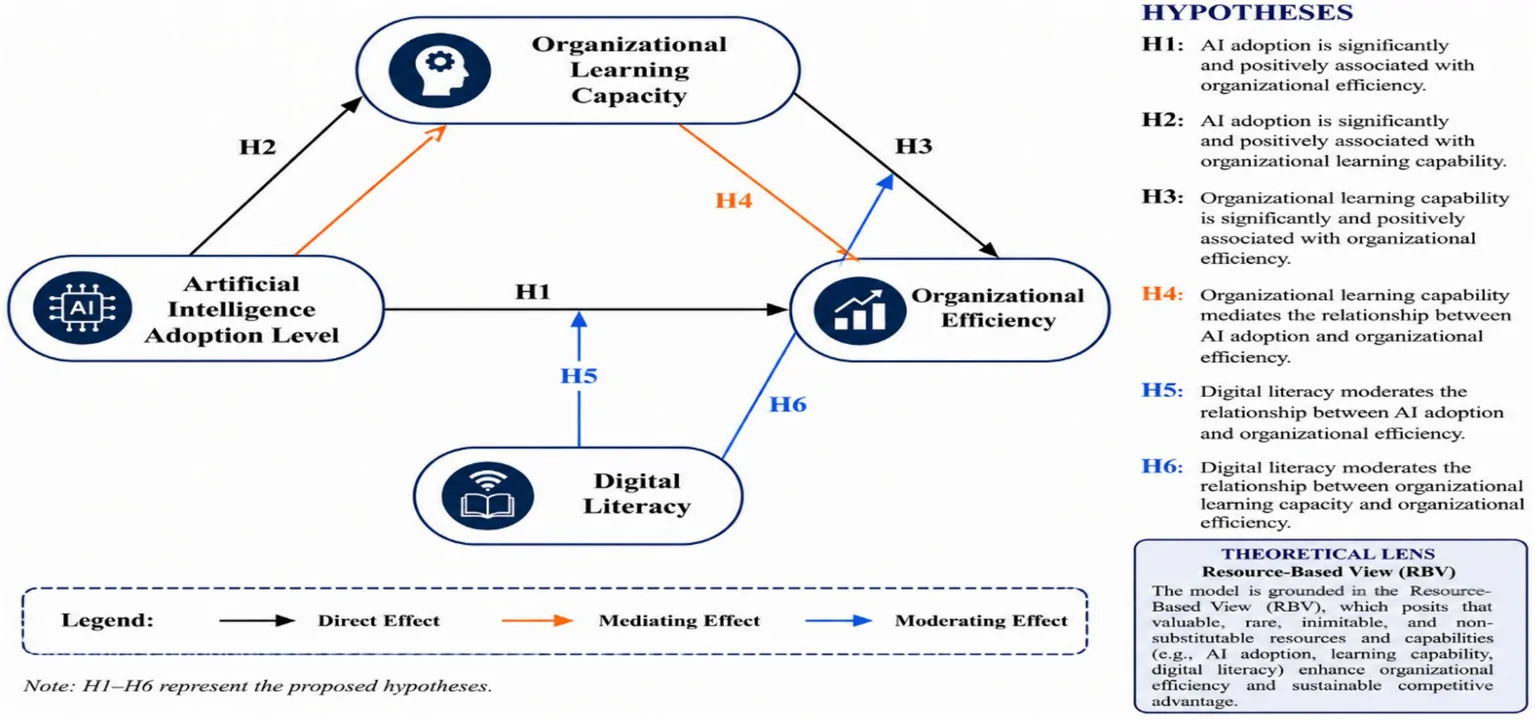
Research model.

## 3 Methodology

### 3.1 Research approach

This research uses a quantitative approach, a cross-sectional survey design, and a positivist framework to investigate the hypothesized associations among AI adoption, organizational efficiency, organizational learning capacity, and digital literacy in Ghana’s banking sector (Amadi, 2023; Obeng, 2023). The research uses a perceptual approach at the employee level, with bank employees as respondents and key informants on organizational phenomena. The study evaluated AI adoption, organizational learning capacity, and organizational efficiency based on employees’ views of organizational methods and outcomes. At the same time, digital literacy encompasses employees’ own digital expertise and competencies pertinent to their roles. The quantitative approach enables methodical evaluation of the study variables, while the cross-sectional survey framework allows data collection at a single point in time to assess proposed correlations. The cross-sectional approach is suitable for analyzing interrelations among the study variables among employees at various financial institutions without altering them. The positivist framework validates the use of standardized instruments and statistical methods to ensure precision, impartiality, and reproducibility of findings. This methodology aligns with empirical research that uses quantitative survey techniques in similar domains (Chirkov and Anderson, 2018; Hair et al., 2017). Self-administered surveys are especially effective for gathering consistent responses from personnel in Ghana’s banking sector, known for its methodical practices and ongoing digital transformation.

### 3.2 Procedure and sampling technique

The 10-times rule, a heuristic for determining the minimum sample size in Partial Least Squares Structural Equation Modeling (PLS-SEM), was used to determine the research sample size (Hair et al., 2017). This standard stipulates that the minimum sample size should be at least ten times the maximum number of indicators assessing the most complex construct or the largest number of structural paths directed at a latent variable. The Organizational Learning Capability construct consisted of sixteen items across four dimensions—managerial commitment, systems perspective, openness and experimentation, and knowledge transfer and integration—making it the most complex construct in the measuring model. Consequently, the suggested minimum sample size was 160 participants.

To enhance the reliability and stability of the PLS-SEM parameter estimates, 500 structured questionnaires were distributed to employees of commercial banks in Ghana. Of these, 393 questionnaires were returned, representing a response rate of 78.6%. After data screening, five questionnaires were excluded because of incomplete responses and response inconsistencies, leaving 388 usable questionnaires and a retention rate of 98.73%. The final usable sample exceeded the suggested minimum for PLS-SEM and was therefore considered sufficient to evaluate the proposed structural model (Hair et al., 2019; Kock and Hadaya, 2018).

The decision to obtain a large sample was moreover influenced by the complexity of the suggested model, which included a mediating relationship via Organizational Learning Capability and a moderating association related to digital literacy. More complex structural models usually necessitate a larger sample size to obtain reliable parameter estimates and reduce the probability of Type II errors (Westland, 2010).

Data collection occurred over 12 weeks from February 16 to May 11, 2026, following ethical approval granted by the relevant institutional review board, Multimedia University, Cyberjaya, Malaysia, and permission obtained from the participating banks in Ghana. Data were gathered from employees across 23 commercial banks operating in Ghana through a multistage sampling method. Regional management initially authorized the study after endorsing the research proposal. In the second phase, regional and human resource (HR) managers worked with branch managers to disseminate questionnaires across participating branches. In the final stage, the researchers used convenience sampling to recruit employees who were available and willing to participate. Individual bank employees served as the unit of analysis. Although AI adoption, organizational learning capacity, and organizational efficiency are organizational phenomena, the researchers assessed them through employees’ perceptions of organizational practices and outcomes. In contrast, digital literacy encompassed employees’ personal digital knowledge and competencies pertinent to their roles. Consequently, responses were retained and examined at the individual level rather than aggregated at the bank or branch level.

To enable data collection across geographically dispersed locations, the research utilized Computer-Assisted Web Interviewing (CAWI). This online survey method reduced interviewer bias, ensured consistent questionnaire presentation, and allowed respondents to complete the survey anonymously and at their convenience.

Despite the potential limitations of convenience sampling on sample representativeness, several strategies were employed to improve sample diversity. Human Resources managers were requested to distribute the survey among employees across various branches, departments, job levels, and functional areas. This approach increased participant heterogeneity and supported examination of the proposed relationship among the study variables.

All participants granted informed consent before engaging in the study. The authors’ university Institutional Review Board approved the study. Participants received an online consent form explaining the study’s purpose, the voluntary nature of participation, and assurances of anonymity and confidentiality. Respondents could proceed with the questionnaire only after providing electronic consent.

### 3.3 Measurement

The research examined four main constructs: AI adoption, organizational efficiency, organizational learning capability, and digital literacy. All constructs were measured using a five-point Likert scale ranging from 1 = strongly disagree to 5 = strongly agree (Leutner et al., 2017; Aletta et al., 2018). The measurement instruments were adapted from established instruments to ensure their applicability to the research’s organizational and banking context.

*AI Adoption scale:* AI adoption was assessed by three items adapted from Cheng et al. (2023). The items evaluate the degree to which the organization has committed resources, participated in, and shown enthusiasm for integrating AI technology to gain a competitive edge. The wording remained at the organizational level by specifically mentioning “this company,” ensuring the metric reflects organizational rather than individual AI adoption.

*Organizational efficiency scale:* Organizational efficiency was measured using an 11-item scale adapted from Atiyyah (1999), as utilized by Sözbilir (2018). The scale captures organizational outcomes relating to workforce and resource utilization, work methods, techniques and procedures, employee competencies, service expansion and quality, service speed and punctuality, responsiveness to clients, and service costs. The items were framed to illustrate the operational activities of the organizations represented by the participants.

*Organizational learning capability scale:* We measured organizational learning capability using 16 items adapted from Jerez-Gomez et al. (2005) across four dimensions: managerial commitment, systems perspective, openness and experimentation, and knowledge transfer and integration. Managerial commitment captures management’s support for employee learning, participation, adaptation, and innovation. Systems perspective assesses employees’ understanding of organizational objectives and the interrelationships among organizational units. Openness and experimentation reflect organizational encouragement of innovation, learning from external sources, and employee suggestions. Knowledge transfer and integration captures the discussion of errors and new ideas, teamwork, and mechanisms for retaining organizational knowledge. Before evaluating the measurement model, we reverse-coded negatively worded items (MC2 and TR3) to ensure consistency across indicators, with higher scores indicating higher levels of the corresponding constructs.

*Digital literacy scale:* Digital literacy was assessed by 10 questions adapted from Ng (2012) and tailored to the professional environment. The measure comprises three dimensions: technical, cognitive, and socio-emotional literacy. Technical literacy evaluates employees’ proficiency in resolving technical issues, acquiring knowledge of emerging technologies, and utilizing ICT competencies in their tasks. Cognitive literacy encompasses employees’ confidence in locating and assessing online material, as well as their understanding of challenges linked to internet activities, such as cybersecurity and information use. Socio-emotional literacy assesses workers’ proficiency in using ICT for collaboration and seeking support for job-related activities through digital communication channels. In adapting the original items, we replaced education-specific references such as university work, learning activities, and friends with workplace-relevant references such as work tasks, colleagues, and work-related activities, while retaining the underlying meaning of the original dimensions.

Both organizational learning capability and digital literacy were specified both as higher-order constructs (HOCs) because of their multidimensional structures. Organizational learning capability was represented by its four dimensions: managerial commitment, systems perspective, openness and experimentation, and knowledge transfer and integration, whereas digital literacy was represented by technical, cognitive, and socio-emotional literacy. The higher-order constructs were assessed utilizing a two-stage methodology, in which the latent variable scores from the corresponding lower-order constructs served as indicators for the higher-order constructs during the second stage of the PLS-SEM analysis.

After finalizing the measurement items, the measurement model was evaluated for reliability and validity. The phrasing of the questions was examined to guarantee clarity and understanding among participants, using generic workplace terms instead of industry-specific jargon where appropriate. Appendix A provides the full measurement tool and the sources from which the items were adapted.

### 3.4 Characteristics of respondents

Table 1 presents the demographic characteristics of the respondents, providing a comprehensive overview of their gender, age, educational attainment, and job position. Among the respondents, 58% were male, and 42% were female. Most respondents (77%) were aged 31 to 50, indicating a workforce that is primarily seasoned and experienced. In terms of educational attainment, 5% held a high school diploma or lower, 62% had a bachelor’s or associate’s degree, and 33% held either a master’s or doctoral degree. By job position, 50% of participants were middle-level managers, 28% were front-line managers, and 22% were senior-level managers.

**Table 1:** Demographic characteristics of respondents.

Characteristics	Category	Frequency (*n*)	Percentage (%)
Gender	Male	225	58.0
	Female	163	42.0
Age	20–30	70	18.0
	31–40	135	34.8
	41–50	165	42.5
	51 or above	18	4.6
Job position in company	Senior-level managers	85	21.9
	Middle-level managers	195	50.3
	Frontline managers	108	27.8
Qualification	High school or below	20	5.2
	Bachelor’s/Associate degree	240	61.9
	Master’s or PhD	128	33.0

### 3.5 Method of analysis

The empirical analysis used partial least squares structural equation modeling (PLS-SEM). The data were first screened and analyzed in SPSS version 23, including checks for missing values, potential outliers, and descriptive statistics. The PLS-SEM evaluations were then conducted with SmartPLS version 4.0.1.8. The analysis proceeded in two stages. First, the measurement model was assessed to determine construct reliability and validity. Internal consistency reliability was evaluated using Cronbach’s alpha and composite reliability (CR), while convergent validity was assessed using outer loadings and average variance extracted (AVE) (Cheung et al., 2024). The heterotrait-monotrait ratio (HTMT) was utilized to evaluate discriminant validity (Henseler et al., 2015).

Because organizational learning capability and digital literacy are higher-order constructs, the higher-order measurement models were assessed using the two-stage method in SmartPLS (Sarstedt et al., 2019). The latent variable scores from the lower-order dimensions served as indicators for the related higher-order constructs in the second stage.

Subsequently, the structural model was evaluated after confirming the measurement model’s adequacy. Collinearity was assessed using variance inflation factor (VIF) scores. The model’s explanatory power was assessed using the coefficient of determination (*R*^2^), while effect sizes (*f*^2^) were used to assess the impact of specific exogenous constructs on the endogenous constructs. Predictive relevance was evaluated using Q^2^. The proposed associations were evaluated via bootstrapping with 5,000 resamples employing two-tailed tests at a 95% confidence level.

We examined the direct effects to test the proposed associations among the constructs. We evaluated mediation by examining the significance and confidence interval of the indirect effect of AI adoption on organizational efficiency via organizational learning capability. The influence of moderation was analyzed through the interaction terms involving digital literacy and the relevant predictor variables. We considered a proposed association supported when its bootstrapped confidence interval excluded zero and the associated *p*-value fell below an established significance threshold (Rovetta et al., 2025). This systematic approach helped establish the measurement model’s reliability and validity before assessing the proposed structural relationships.

## 4 Results and findings

### 4.1 Measurement model and preliminary results

The measurement model was initially evaluated to assess the reliability and validity of the constructs before analyzing the structural relationships. The overall model was evaluated with the Standardized Root Mean Square Residual (SRMR) and the Normed Fit Index (NFI). According to Table 2, the saturated model showed an SRMR of 0.056 and an NFI of 0.900 (Wah, 2025), indicating adequate model fit and a relatively small divergence between observed and model-implied correlations.

**Table 2:** Measurement model fitness.

Indices	Values	Rating
SRMR	0.056	Good fit
NFI	0.900	Adequate

#### 4.1.1 Reliability and validity analysis

The measurement model’s reliability and validity was evaluated at both the lower-order construct (LOC) and higher-order construct (HOC) levels. Indicator reliability was assessed using standardized outer loadings, while internal consistency reliability was determined using Cronbach’s alpha (α) and composite reliability (CR). Convergent validity was evaluated using the average variance extracted (AVE), following established PLS-SEM methods (Hair et al., 2020).

Indicator reliability reflects the extent to which an observed indicator adequately represents its underlying construct (Sarstedt et al., 2019). Outer loadings of 0.70 or higher are usually considered acceptable; nonetheless, indicators with loadings ranging from 0.40 to 0.70 may be retained if their exclusion does not significantly enhance the construct’s reliability or convergent validity, provided there is adequate theoretical rationale for their retention (Hair et al., 2020; Shrestha, 2021). Table 3 shows that the standardized indicator loadings at the LOC level ranged from 0.639 to 0.978 (see Figure 2). The indicators with loadings below 0.70 were retained because the related constructs showed satisfactory internal consistency reliability and convergent validity, and the indicators retained theoretical relevance to their respective constructs.

**Table 3:** Reliability and convergence validity results (LOC).

Construct and items	Standardized loadings	Cronbach’s alpha	CR	AVE
Artificial intelligence adoption				
This company has invested funds in AI technology (AIAD1)	0.942	0.944	0.946	0.900
This company has been involved in the adoption of AI technology (AIAD2)	0.961			
This company has been interested in adopting AI technology to gain a competitive advantage (AIAD3)	0.943			
Digital literacy				
I am confident in my ability to search for and evaluate information obtained from the Web for work-related purposes (COGN1)	0.968	0.931	0.967	0.936
I am familiar with issues related to web-based activities, such as cybersecurity, online search, and information use (COGN2)	0.967			
ICT enables me to collaborate better with my colleagues on work projects and other work-related activities (SE1)	0.978	0.954	0.977	0.956
I frequently obtain help with my work tasks from my colleagues through the Internet and other digital communication platforms (SE2)	0.977			
I know how to solve my own technical problems when using digital technologies at work (TECH1)	0.961	0.978	0.982	0.901
I can learn new digital technologies easily (TECH2)	0.935			
. I keep up with important new digital technologies relevant to my work (TECH3)	0.957			
I know about a wide range of digital technologies (TECH4)	0.955			
. I have the technical skills I need to use ICT for my work and to create digital materials relevant to my job (TECH5)	0.948			
I have good ICT skills for performing my work (TECH6)	0.938			
Organizational learning capability				
The managers frequently involve their staff in important decision-making processes (MC1)	0.936	0.954	0.964	0.844
Employee learning is considered more of an expense than an investment. (MC2)	0.894			
The firm’s management looks favorably on carrying out changes in any area to adapt to and/or keep ahead of new environmental situations (MC3)	0.909			
Employee learning capability is considered a key factor in this firm (MC4)	0.927			
In this firm, innovative ideas that work are rewarded (MC5)	0.926			
All employees have generalized knowledge regarding this firm’s objectives (SP1)	0.872	0.884	0.928	0.812
All parts that make up this firm (departments, sections, work teams, and individuals) are aware of how they contribute to achieving the overall objectives (SP2)	0.920			
All parts that make up this firm are interconnected and work together in a coordinated fashion (SP3)	0.912			
This firm promotes experimentation and innovation as a way of improving work processes (EX1)	0.876	0.859	0.905	0.703
This firm follows what other firms in the sector are doing and adopts practices and techniques it considers useful and relevant (EX2)	0.807			
sources (e.g., advisors, customers, training firms) are considered useful for this firm’s learning (EX3)	0.828			
Part of this firm’s culture is that employees can express their opinions and make suggestions regarding the procedures and methods used to carry out tasks (EX4)	0.841			
Errors and failures are always discussed and analyzed in this firm at all levels (TR1)	0.915	0.926	0.947	0.819
Employees have the opportunity to discuss new ideas, programs, and activities among themselves that might be useful to the firm (TR2)	0.929			
In this firm, teamwork is not the usual way of working (TR3)	0.936			
The firm has instruments (e.g., manuals, databases, files, and organizational routines) that allow what has been learned from past situations to remain valid, even when employees change (TR4)	0.836			
Organizational efficiency				
Utilization of manpower (OREFF1)	0.738	0.909	0.924	0.526
Improvement in work methods (OREFF2)	0.766			
Improvement in techniques and procedures over the past 3 years (OREFF3)	0.722			
Development of employees’ competencies and skills over the past 3 years (OREFF4)	0.744			
Expansion of services achieved over the past 3 years (OREFF5)	0.776			
Improvement in the quality of services over the past 3 years (OREFF6)	0.777			
Speed of providing services (OREFF7)	0.722			
Punctuality in providing services (OREFF8)	0.662			
Responsiveness to clients’ needs (OREFF9)	0.715			
Utilization of available resources (OREFF10)	0.639			
Cost of services (OREFF11)	0.703			

AIAD, artificial intelligence adoption; DL, digital literacy; COGN, cognitive literacy; SE, socio-emotional literacy; TECH, technical literacy; OLC, organizational learning capability; MC, managerial commitment; SP, systems perspective; EX, openness and experimentation; TR, knowledge transfer and integration; OREFF, organizational efficiency; α, Cronbach’s alpha; CR, composite reliability; AVE, average variance extracted. MC2 and TR3 were reverse-coded before analysis. DL and OLC are higher-order constructs, while AIAD and OREFF are first-order constructs.

**Figure 2:**
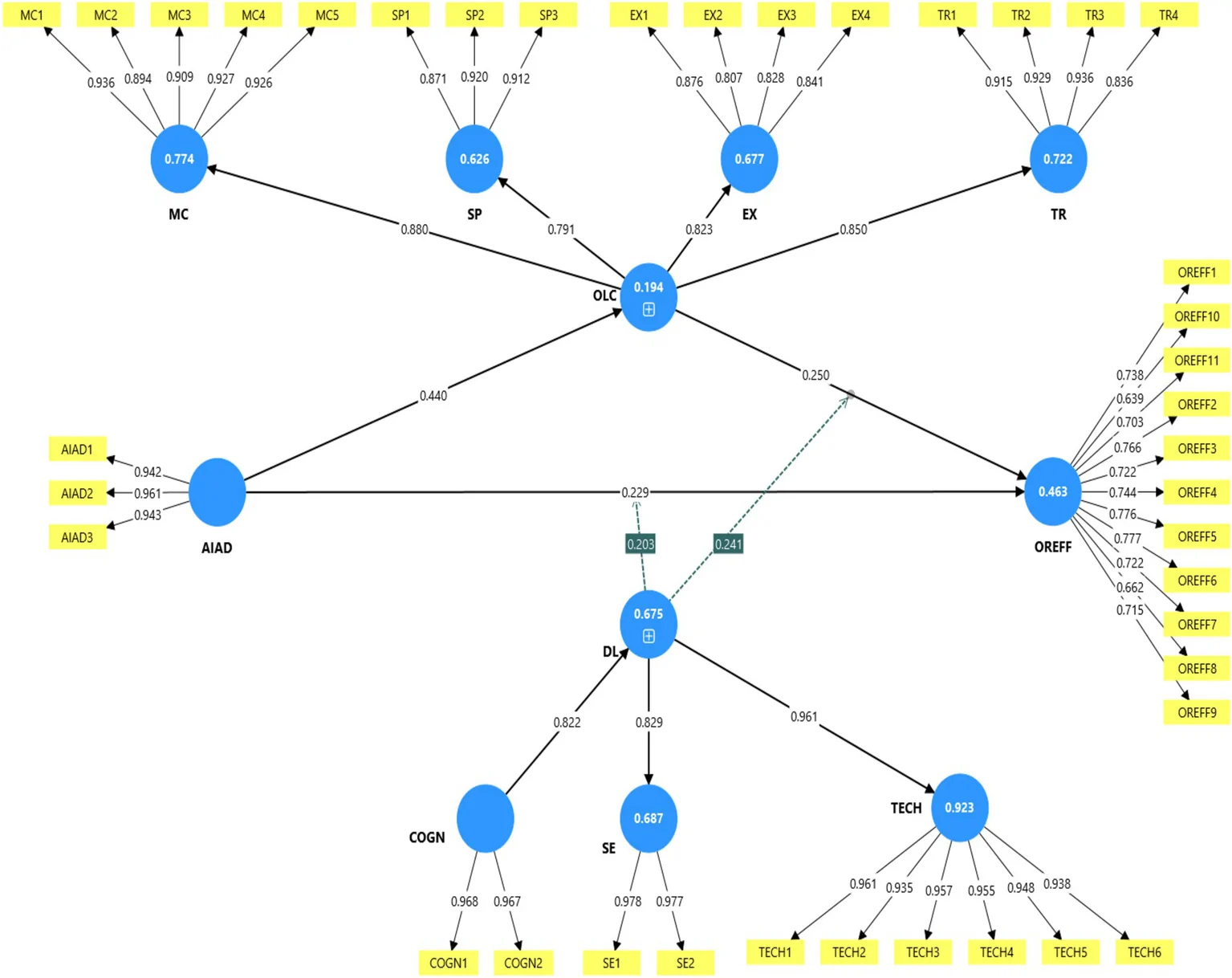
Measurement model (LOC loadings).

Internal consistency reliability was evaluated using Cronbach’s alpha and composite reliability. Scores of 0.70 or above are usually considered indicative of acceptable internal consistency reliability (Hair et al., 2020). At the LOC level (see Table 3), Cronbach’s alpha values varied between 0.859 and 0.978, whereas CR values varied from 0.905 to 0.982. At the HOC level, Cronbach’s alpha coefficients varied between 0.860 and 0.944, whereas CR values varied from 0.905 to 0.964 (Table 4). All values exceeded the suggested limit, indicating adequate internal consistency.

**Table 4:** Reliability and convergence validity results (HOC).

Construct	HOC loading	α	CR	AVE
Artificial intelligence adoption (AIAD)	–	0.944	0.964	0.900
Digital literacy				
COGN	0.873	0.867	0.918	0.789
SE	0.896			
TECH	0.895			
Organizational learning capability				
MC	0.855	0.860	0.905	0.703
SP	0.810			
EX	0.834			
TR	0.854			
Organizational efficiency (OREFF)	–	0.909	0.924	0.526

HOC, higher-order construct; HOC loadings represent the loadings of the lower-order dimensions on their respective higher-order constructs. A dash (–) indicates that the construct is first-order and therefore has no HOC loading.

Content validity was confirmed via expert assessment of the questionnaire items. The professional evaluation considered the pertinence, clarity, comprehensiveness, and correspondence of the items with their intended constructs (Lim, 2024; Asare Obeng and Atan, 2025). This evaluation was especially significant as established measurement tools were contextually modified to align with the organizational and working environment of the research while preserving the underlying meaning of the original measures.

The evaluation of convergent validity was conducted using the average variance extracted AVE. An AVE score of 0.50 or above indicates that a construct accounts for at least 50% of the variance in its indicators and is generally considered indicative of acceptable convergent validity (Hair et al., 2020). At the LOC level, AVE values ranged from 0.526 to 0.956 (see Table 3). At the HOC level, AVE values ranged from 0.526 to 0.900 (Table 4). All AVE values exceeded the suggested threshold of 0.50, indicating acceptable convergent validity.

At the HOC level, the lower-order dimensions exhibited significant loadings on their corresponding higher-order constructs. The loadings for digital literacy were 0.873 for cognitive literacy, 0.896 for socio-emotional literacy, and 0.895 for technical literacy (see Figure 3). The loadings for organizational learning capability were 0.855 for managerial commitment, 0.810 for systems perspective, 0.834 for openness and experimentation, and 0.854 for knowledge transfer and integration. These findings suggest that the lower-order dimensions significantly contribute to their corresponding higher-order constructs.

**Figure 3:**
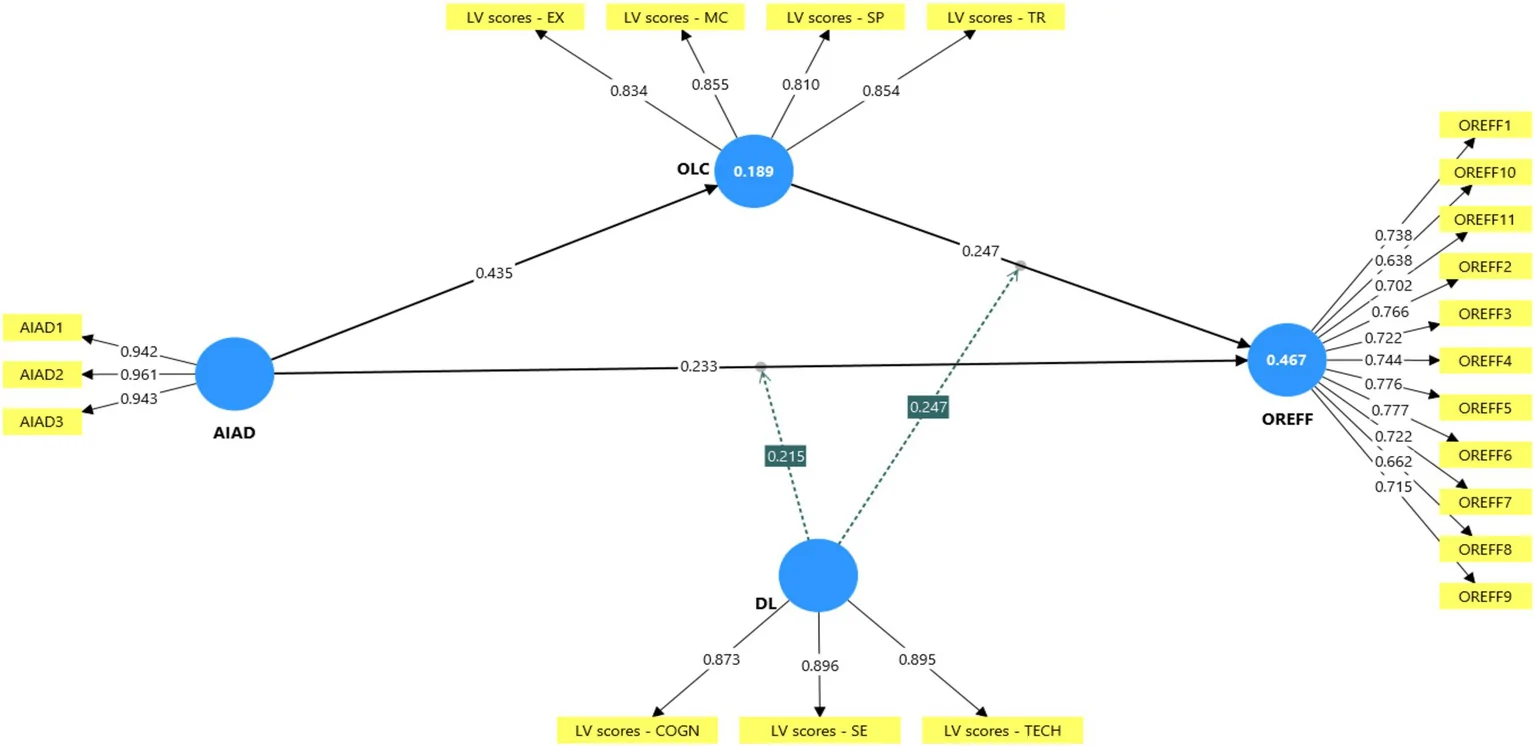
HOC loadings.

The findings demonstrate adequate indicator reliability, internal consistency, and convergent validity at both the LOC and HOC tiers. The results thus validate the measurement model for subsequent evaluation of the structural relationships.

#### 4.1.2 Discriminant validity

After evaluating indicator reliability, internal consistency, and convergent validity, discriminant validity was assessed using the heterotrait–monotrait (HTMT) correlation ratio. The HTMT criterion is recommended for evaluating discriminant validity in PLS-SEM because it has proven more reliable than conventional methods such as the Fornell–Larcker criterion and cross-loadings (Henseler et al., 2015). A conservative benchmark of 0.85 was established, whereby HTMT values falling below 0.85 signify adequate discriminant validity among conceptually distinct constructs (Henseler et al., 2015).

According to Table 5, the HOC-level HTMT values for AI adoption, digital literacy, organizational learning capability, and organizational efficiency ranged from 0.414 to 0.500. The HTMT values were 0.427 for AI adoption and digital literacy, 0.477 for AI adoption and organizational learning capability, 0.468 for AI adoption and organizational efficiency, 0.414 for digital literacy and organizational learning capability, 0.493 for digital literacy and organizational efficiency, and 0.500 for organizational learning capability and organizational efficiency. All values are far below the cautious 0.85 benchmark, indicating that the four main constructs are empirically separate and that discriminant validity is confirmed.

**Table 5:** Discriminant validity assessment (HOC-HTMT).

Construct	AIAD	DL	OLC	OREFF
AIAD				
DL	0.427			
OLC	0.477	0.414		
OREFF	0.468	0.493	0.500	

### 4.2 Structural model

Before evaluating the proposed relationships, we assessed the structural model for common method bias, multicollinearity, *f*^2^, *R*^2^, and *Q*^2^predict. The evaluation included Harman’s single-factor test, the inner variance inflation factor, *f*^2^, *R*^2^, and *Q*^2^predict.

#### 4.2.1 Common method bias

Because the research is based on self-reported survey data, we evaluated the potential for CMB using two complementary methods. First, Harman’s single-factor analysis was conducted using principal axis factoring to determine whether a single factor explained a significant amount of the variance among the measurement variables. According to Appendix B, the first factor represented 36.363% of the total variation, falling below the common 50% benchmark, indicating that one factor does not dominate the variance (Podsakoff et al., 2003). Second, we analyzed the inner VIF metrics. Table 6 illustrates that the VIF values varied from 1.000 to 1.339, well below the cautious limit of 3.3 suggested by Kock (2015). These findings reveal no evidence of significant common method bias or concerning multicollinearity within the structural model.

**Table 6:** Effect size and VIF results.

Path	*f* ^2^	VIF
Artificial intelligence adoption → Organizational learning capability	0.233	1.000
Artificial intelligence adoption → Organizational efficiency	0.076	1.339
Digital literacy → Organizational efficiency	0.108	1.241
Organizational learning capability → Organizational efficiency	0.088	1.302
Digital literacy x Artificial intelligence adoption → Organizational efficiency	0.065	1.201
Digital literacy x Organizational learning capability → Organizational efficiency	0.091	1.198

#### 4.2.2 Effect size

The effect size (*f*^2^) was analyzed to assess each predictor’s impact on the explained variance of the endogenous constructs (Cohen, 2013). Table 6 shows that AI adoption had an *f*^2^ of 0.233 in forecasting organizational learning capability, indicating a moderate impact. For organizational efficiency, AI implementation had a modest impact (*f*^2^ = 0.076), while digital literacy showed a somewhat greater benefit (*f*^2^ = 0.108). The capacity for organizational learning exerted a minor influence on organizational efficiency (*f*^2^ = 0.088). The interaction effects of digital literacy and AI adoption (*f*^2^ = 0.065), as well as digital literacy and organizational learning capability (*f*^2^ = 0.091), also indicated minor effects.

#### 4.2.3 Predictive relevance

The *Q*^2^predict procedure was used to assess predictive significance. Positive *Q*^2^predict values suggest that the model possesses predictive significance for the endogenous constructs (Geisser, 1974; Stone, 1974). Table 7 reported *Q*^2^predict values of 0.184 for organizational learning capability and 0.345 for organizational efficiency, indicating that the model has predictive significance for both endogenous constructs. The RMSE and MAE values for organizational learning capability were 0.907 and 0.757, whereas for organizational efficiency, they were 0.813 and 0.654, respectively.

**Table 7:** Coefficient of determination (*R*^2^) and predictive relevance (*Q*^2^) results.

Variables	*R* ^2^	*Q* ^2^	RMSE	MAE
Organizational learning capability	0.189	0.184	0.907	0.757
Organizational efficiency	0.467	0.345	0.813	0.654

#### 4.2.4 Explanatory power

The *R*^2^ (see Table 7) was evaluated to ascertain the extent of variance in each endogenous construct explained by its predictors (Cohen, 2013). The model accounted for 18.9% of the variability in organizational learning capability (*R*^2^ = 0.189) via AI adoption. For organizational efficiency, the predictors jointly accounted for 46.7% of the variation (*R*^2^ = 0.467). Considering that organizational efficiency is predicted by various factors such as AI adoption, digital literacy, organizational learning capability, and the two interaction variables, its *R*^2^ is unlikely to correspond to the square of any individual path coefficient.

Ultimately, we evaluated the structural associations by bootstrapping with 5,000 resamples. The bootstrap findings, including path coefficients, standard errors, t-statistics, *p*-values, and confidence intervals, are shown in the structural model results tables. The analysis used a two-tailed test at the 95% confidence level to assess the statistical significance of the proposed associations.

### 4.3 Hypothesis testing

Once the measurement and structural models met the required assessment criteria, we conducted hypothesis testing using PLS-SEM in SmartPLS with 5,000 bootstrap resamples (Cheah et al., 2026). The analysis examined three direct relationships (H1–H3), the mediating role of organizational learning capability (H4), and two moderating relationships involving digital literacy (H5–H6). We assessed statistical significance using a two-tailed test at the 5% significance level, using 95% bootstrap confidence intervals as an additional criterion for evaluating the hypothesized relationships. Figure 4 presents the estimated structural model and summarizes the direct, mediating, and moderating relationships examined in the study; Table 8 reports the detailed hypothesis-testing results.

**Figure 4:**
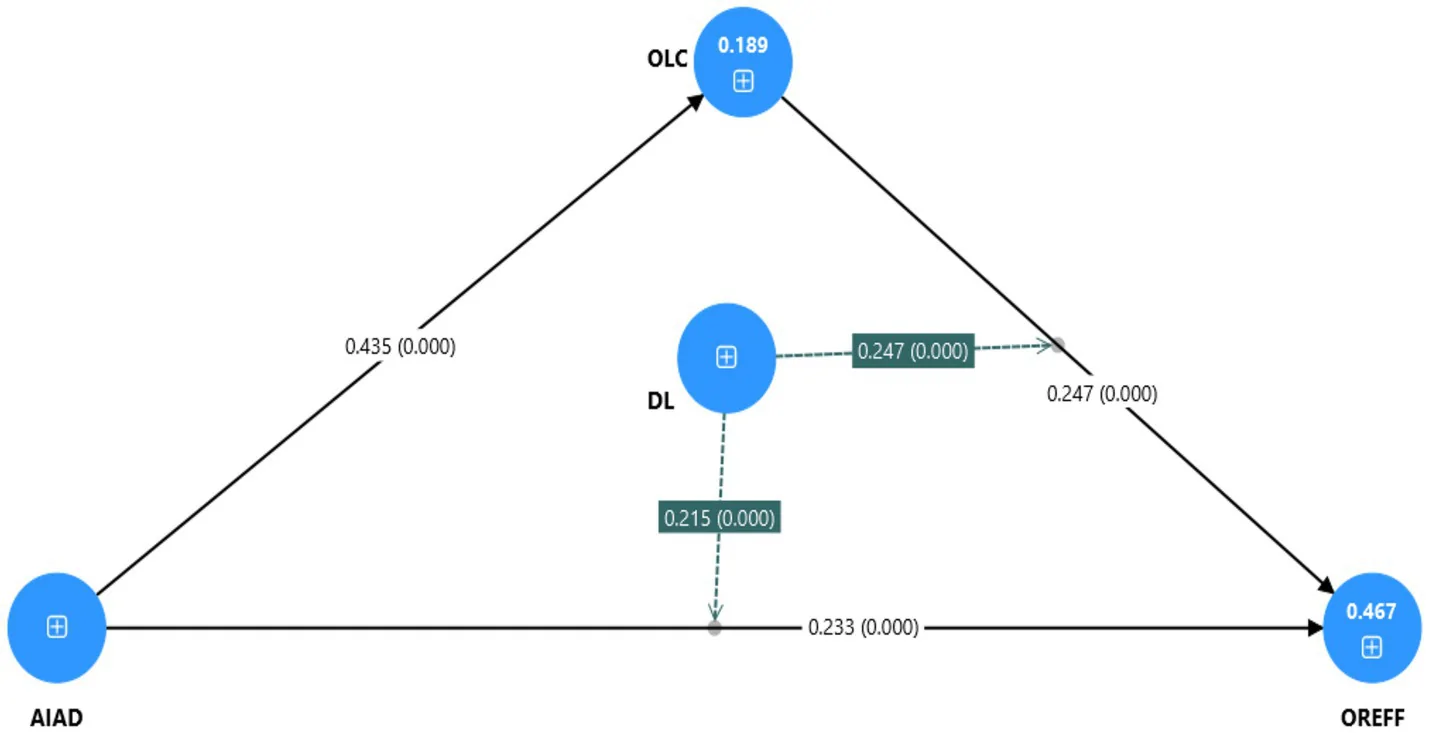
Structural model. Values shown represent standardized path coefficients; values in parentheses represent *p*-values.

**Table 8:** Hypotheses test results.

Hypothesis	Path	*β*	STDEV	*t*-value	*p*-value	95% CI	Decision
H1	AIAD → OREFF	0.233	0.046	5.030	<0.001	[0.142, 0.325]	Supported
H2	AIAD → OLC	0.435	0.041	10.516	<0.001	[0.352, 0.516]	Supported
H3	OLC → OREFF	0.247	0.044	5.659	<0.001	[0.160, 0.333]	Supported
–	DL → OREFF	0.267	0.045	5.968	<0.001	[0.181, 0.355]	–
H4	AIAD → OLC → OREFF	0.107	0.021	4.990	<0.001	[0.068, 0.154]	Supported
H5	DL × AIAD → OREFF	0.215	0.044	4.875	<0.001	[0.124, 0.298]	Supported
H6	DL × OLC → OREFF	0.247	0.041	6.051	<0.001	[0.164, 0.324]	Supported

*β*, standardized path coefficient; STDEV, standard deviation; CI, confidence interval; *p*-values are based on 5,000 bootstrap resamples; a hypothesis is considered supported when the 95% confidence interval does not include zero.

#### 4.3.1 Direct effects

H1 proposed that AI adoption positively influences organizational efficiency. The results show a positive and statistically significant effect of AI adoption on organizational efficiency (*β* = 0.233, *t* = 5.030, *p* < 0.001, 95% CI [0.142, 0.325]). Because the confidence interval does not include zero, the results support H1.

H2 proposed that AI adoption positively influences organizational learning capability. The results indicate a positive and significant relationship (*β* = 0.435, *t* = 10.516, *p* < 0.001, 95% CI [0.352, 0.516]). Thus, greater AI adoption is associated with higher organizational learning capability, supporting H2.

H3 proposed a positive relationship between organizational learning capability and organizational efficiency. The results show a positive and significant effect (*β* = 0.247, *t* = 5.659, *p* < 0.001, 95% CI [0.160, 0.333]). Therefore, H3 is supported.

In addition to the hypothesized relationships, the structural model revealed a significant positive main effect of digital literacy on organizational efficiency (*β* = 0.267, *t* = 5.968, *p* < 0.001, 95% CI [0.181, 0.355]). The effect size was *f*^2^ = 0.108, indicating a small-to-moderate contribution to organizational efficiency. This finding indicates that, independent of the interaction effects, higher levels of employees’ digital literacy are associated with higher organizational efficiency.

#### 4.3.2 Mediating effect

H4 examined whether organizational learning capability mediates the relationship between AI adoption and organizational efficiency. We assessed mediation using the bootstrapped indirect effect, with AI adoption as the predictor, organizational learning capability as the mediator, and organizational efficiency as the outcome. The specific indirect effect of AI adoption on organizational efficiency through organizational learning capability was positive and significant (*β* = 0.107, *t* = 4.990, *p* < 0.001, 95% CI [0.068, 0.154]). Because the confidence interval excludes zero, the indirect effect is statistically significant, supporting H4.

The direct effect of AI adoption on organizational efficiency also remained positive and significant (*β* = 0.233, *p* < 0.001). Accordingly, the mediation is partial rather than full. The variance accounted for (VAF) was approximately 31.5%, calculated as the indirect effect divided by the total effect (indirect effect + direct effect). Thus, organizational learning capability accounts for part of the relationship between AI adoption and organizational efficiency, while AI adoption retains a direct effect on organizational efficiency.

#### 4.3.3 Moderating effects

We assessed the moderating role of digital literacy in SmartPLS by specifying the corresponding interaction relationships between digital literacy and the predictor constructs (digital literacy × AI adoption and digital literacy × organizational learning capability). We evaluated the significance of the interaction effects using bootstrapping with 5,000 resamples and a 95% confidence interval.

For H5, the interaction between digital literacy and AI adoption had a positive and statistically significant effect on organizational efficiency (*β* = 0.215, *t* = 4.875, *p* < 0.001, 95% CI [0.124, 0.298]). Because the confidence interval does not include zero, H5 was supported. As shown in Figure 5, the positive relationship between AI adoption and organizational efficiency strengthens as digital literacy increases from a low level (−1 SD) to a high level (+1 SD). This indicates that higher digital literacy strengthens the positive effect of AI adoption on organizational efficiency.

**Figure 5:**
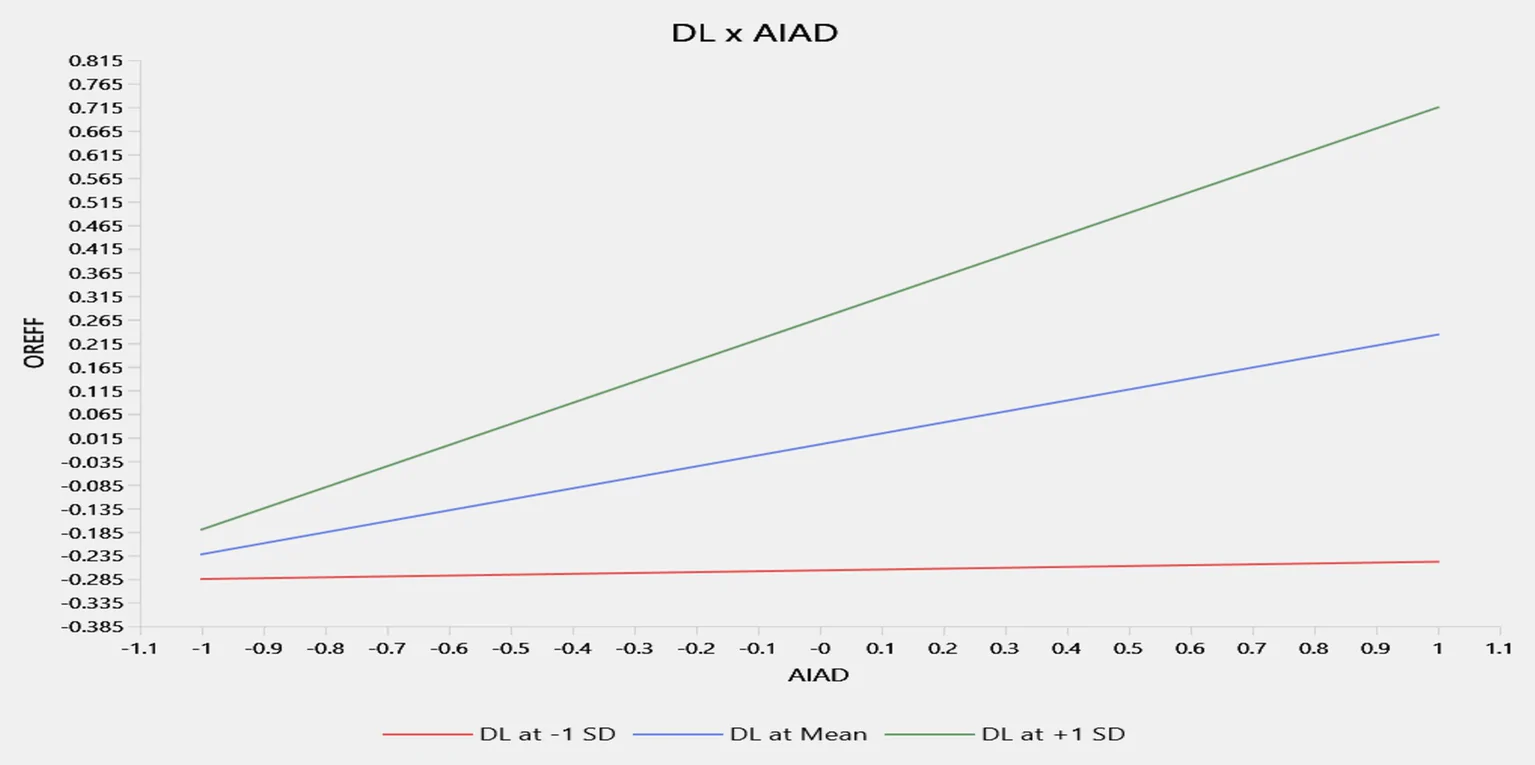
Moderating effect of digital literacy on the relationship between AI adoption and organizational efficiency.

Similarly, H6 proposed that digital literacy moderates the relationship between organizational learning capability and organizational efficiency. The interaction effect was positive and statistically significant (*β* = 0.247, *t* = 6.051, *p* < 0.001, 95% CI [0.164, 0.324]), supporting H6. As shown in Figure 6, the positive relationship between organizational learning capability and organizational efficiency strengthens at higher levels of digital literacy. Thus, digital literacy positively strengthens the effect of organizational learning capability on organizational efficiency.

**Figure 6:**
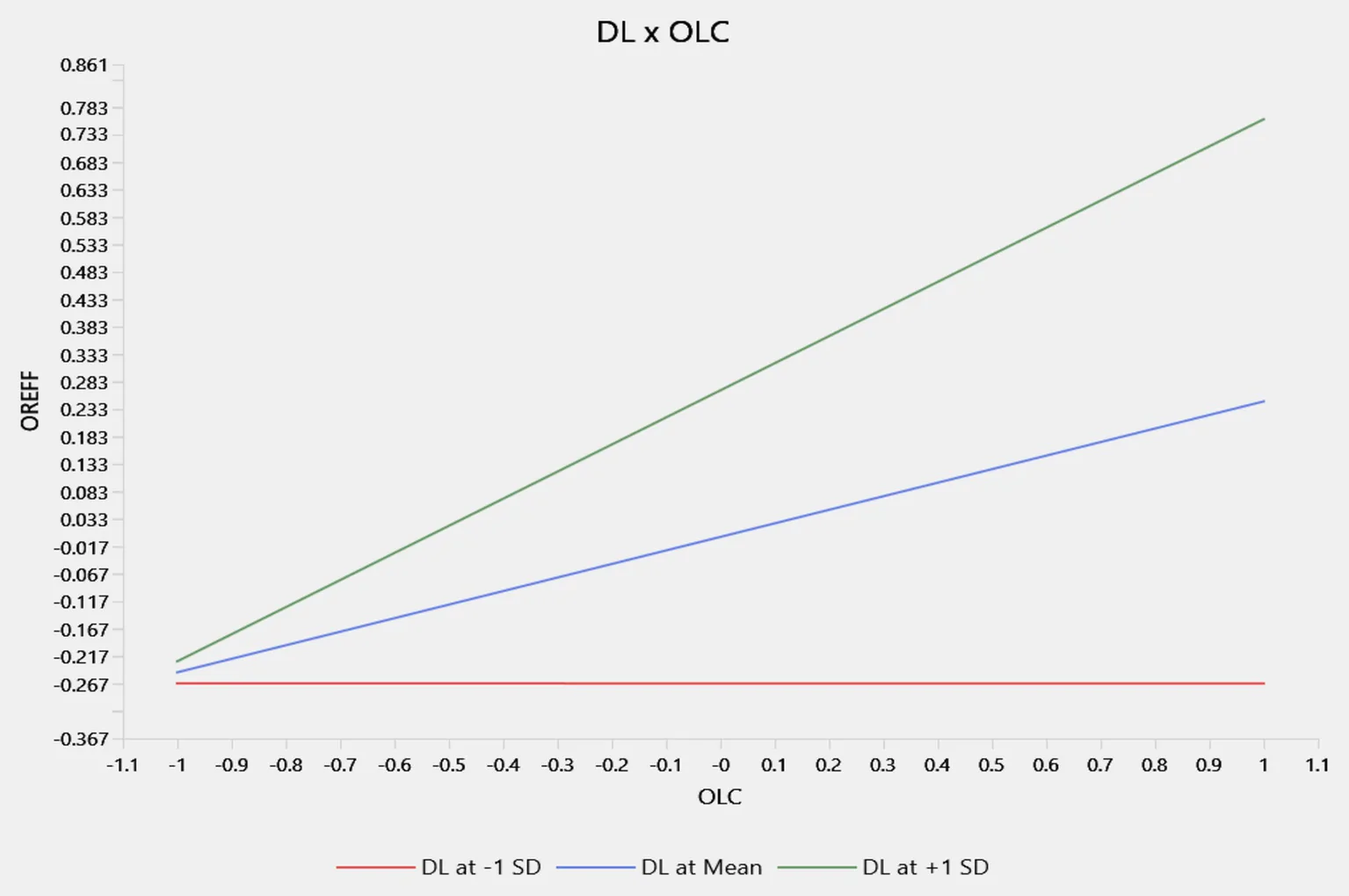
Moderating effect of digital literacy on the relationship between organizational learning capability and organizational efficiency.

Overall, all six hypotheses are supported. The findings indicate that AI adoption positively influences organizational learning capability and organizational efficiency, while organizational learning capability positively contributes to organizational efficiency. Organizational learning capability also partially mediates the relationship between AI adoption and organizational efficiency. Furthermore, digital literacy positively strengthens both the AI adoption–organizational efficiency and organizational learning capability–organizational efficiency relationships.

## 5 Discussion

This research found a significant, positive association between AI adoption and organizational efficiency. This suggests that Ghanaian banks adopting AI technologies to automate customer service, detect fraud, and perform data analytics can significantly improve their operational processes and overall efficiency. These findings align with recent empirical research showing that AI adoption improves organizational efficiency through stronger process automation, support for data-driven decision-making, and improved organizational responsiveness (Dwivedi et al., 2021; Huang and Rust, 2021; Kshetri, 2024). This further supports Yang (2022), who found that companies adopting AI outperform competitors in productivity and operational efficiency. This favorable association suggests that AI serves as a strategic resource for organizations, augmenting efficiency when effectively integrated into organizational processes, aligning with the Resource-Based View (Barney, 1991). These results indicate that the organizational value of AI transcends mere technological automation, enhancing operational effectiveness in Ghanaian banks.

The results also indicate that AI adoption was positively and significantly associated with organizational learning capability. This suggests that adopting AI technologies is associated with improved capacity in Ghanaian banks to acquire, share, and use knowledge more efficiently, thereby strengthening organizational learning mechanisms. These findings align with contemporary research indicating that AI enhances knowledge creation, organizational learning, and continuous innovation by enabling organizations to convert large volumes of data into practical insights for strategic decision-making (Dwivedi et al., 2021; Jöhnk et al., 2021; Kshetri, 2024). The finding further corroborates previous research indicating that intelligent technologies augment organizational learning through enhanced information processing, knowledge sharing, and adaptive capabilities. Consistent with the Resource-Based View (Barney, 1991), the findings indicate that AI is a significant technological asset, with its benefits amplified by the cultivation of organizational learning capacities, allowing companies to adapt more effectively to evolving market conditions.

The results additionally demonstrate that organizational learning capability is significantly and positively associated with organizational efficiency. This indicates that banks with strong capabilities to acquire, share, and use knowledge are better able to improve operational processes, enhance decision-making, and adapt to fluctuating market dynamics. This finding aligns with prior empirical research indicating that organizational learning capacity improves efficiency, innovation, and overall performance by facilitating continuous knowledge acquisition and adaptation within the organization (Alegre and Chiva, 2008; Dukhaykh and Alangri, 2026). The findings further corroborate the notion that organizations fostering a strong learning culture are better able to convert knowledge into improved operational outcomes. According to the Resource-Based View (Barney, 1991), Organizational learning capability is a significant organizational asset that enhances technology resources, therefore strengthening an organization’s capacity for greater efficiency (Rashid and Rasheed, 2026).

The results indicate that organizational learning capability partially mediates the relationship between AI adoption and organizational efficiency. This suggests that while the use of AI is directly associated with enhanced organizational efficiency, part of its impact is achieved via improved organizational learning capability. AI technologies enhance organizational value by promoting knowledge acquisition, enabling information sharing, and using organizational knowledge to improve operational processes. This finding aligns with earlier research indicating that technological investments contribute to better organizational outcomes when supported by effective organizational learning processes that convert technological assets into operational capabilities (Garvin et al., 2008; Jöhnk et al., 2021). The complementary partial mediation implies that organizational learning capability is a significant, but not the only, mechanism by which AI adoption improves organizational efficiency, suggesting that additional organizational factors may also play a role in this relationship. These findings align with the Resource-Based View (Barney, 1991), which highlights that technological assets enhance organizational value when complemented by valuable organizational capabilities.

The results suggest that digital literacy strengthens the positive association between AI adoption and organizational efficiency. Employees with stronger digital knowledge and skills are more likely to engage with AI-driven systems, analyze technology-derived data, and integrate intelligent technologies into their routine tasks. Thus, digital literacy helps employees convert technological information into concrete efficiency gains. This viewpoint aligns with studies highlighting the importance of employee digital literacy in achieving organizational efficiency in digital transformation (Vial, 2019; Verhoef et al., 2021; Dwivedi et al., 2021). The finding also indicates that investing in AI technology alone may not create organizational value when employees lack the skills to use it effectively. According to the Resource-Based View (Barney, 1991), digital literacy is an auxiliary human capability that allows businesses to derive greater value from their technological resources.

Moreover, digital literacy enhances the extent to which an organization’s learning capability can be converted into organizational efficiency. Employees with stronger digital skills can more effectively retrieve, assess, share, and use information through digital platforms and technologies (Molla et al., 2025), thereby turning organizational learning into improved work processes and efficiency outcomes. This underscores a significant interdependent connection between organizational learning capacity and digital literacy: learning capabilities enable the organization to acquire and develop knowledge, whereas digital literacy enables employees to interact with and apply that knowledge in increasingly digital workplaces. From a Resource-Based View (Barney, 1991), this finding indicates that organizational capabilities provide greater value when supported by ‘complementary employee skills’. Consequently, enhancing employees’ digital literacy alongside organizational learning capability can help organizations more effectively transform learning and knowledge assets into increased efficiency (Rahman et al., 2026).

## 6 Theoretical implications

This study presents several significant theoretical contributions to the literature on AI, organizational learning, and organizational performance. First, the research extends the Resource-Based View by demonstrating that AI adoption alone does not enhance organizational efficiency. The results indicate that AI generates enhanced organizational value when integrated with significant organizational capabilities, especially organizational learning capability and digital literacy. This supports the Resource-Based View assertion that a competitive advantage is derived from the effective integration of tangible technological resources with valuable intangible organizational capabilities (Barney, 1991).

Secondly, the research contributes to the AI literature by empirically substantiating Organizational Learning Capability as a crucial mechanism that facilitates the enhancement of organizational efficiency through AI adoption. The significant partial mediation effect indicates that companies gain enhanced benefits from AI when technological investments are converted into knowledge acquisition, knowledge sharing, and continuous organizational learning. This research builds upon previous studies by explaining not just whether AI enhances organizational efficiency but also the mechanisms through which these enhancements occur in organizational contexts.

Third, the research enhances the growing literature on digital transformation by identifying digital literacy as a crucial boundary condition in the association between AI adoption and organizational efficiency. The significant moderating effects suggest that the advantages of AI for organizations are enhanced when employees have the necessary digital skills to engage proficiently with AI-driven technology. This finding expands existing theoretical knowledge by highlighting that human capabilities are crucial for unlocking the full organizational benefits of AI investments.

Finally, the research enhances the literature on AI and organizational management by offering empirical data from Ghana’s banking sector, a context often overlooked in underdeveloped countries. Prior research has largely focused on AI adoption in developed nations; however, the results of this study indicate that integrating AI, organizational learning capability, and digital literacy is essential for enhancing organizational efficiency in emerging economies. This broadens the contextual relevance of Resource-Based View in explaining AI-driven organizational transformation across various institutional environments.

## 7 Practical implications

This research provides significant practical insights for policymakers, banking managers, human resource executives, and technology strategists aiming to expedite AI-driven digital transformation in the banking industry. The positive association between AI adoption and organizational efficiency indicates that banks should strategically integrate AI technologies into core operational activities, such as customer service, fraud detection, credit assessment, and decision-making support systems. Instead of perceiving AI as an independent technological investment, organizations should integrate AI into broader organizational strategy focused on enhancing operational efficiency and service quality.

The results additionally show that Organizational Learning Capability has a significant mediating function in converting AI adoption into organizational efficiency. Consequently, managers should enhance AI implementation with strategies that strengthen knowledge acquisition, sharing, and continuous organizational learning. Implementing consistent AI training, interdisciplinary knowledge-sharing platforms, and continuous professional development initiatives can enhance employees’ ability to use AI technology and improve organizational performance.

The significant moderating role of digital literacy underscores the need to enhance employees’ digital skills alongside technological investments. Organizations should emphasize continuous digital skills development to ensure that employees have the necessary competencies to effectively interpret, utilize, and apply AI-driven technologies in their routine tasks. Investments in AI are more likely to yield organizational advantages when complemented by workforce development programs that enhance digital literacy across various organizational levels.

Beyond Ghana, these results offer valuable perspectives for financial institutions and organizations operating in other developing nations where AI adoption is progressing amidst comparable institutional, technological, and workforce limitations.

Policymakers and organizational leaders should advocate for cohesive digital transformation strategies that simultaneously invest in AI technologies, organizational learning, and employee digital skills to optimize the organizational benefits of AI adoption.

The results indicate that sustainable improvements in organizational efficiency require a holistic strategy that complements technological investments with organizational learning and digitally competent employees. An integrated strategy is more likely to support effective AI implementation and long-term organizational performance.

## 8 Limitations and directions for future studies

This study has several limitations that should be acknowledged while interpreting its results. First, despite using a multistage sampling technique, the final stage relied on convenience sampling, potentially introducing selection bias and limiting the sample’s representativeness. Future research should incorporate probability-based sampling methods, including stratified random sampling, to improve the representativeness of samples among various categories of bank employees.

Secondly, the research used a cross-sectional research design, gathering data at one specific point in time. Consequently, the findings should be interpreted as evidence of relationships among the study variables rather than causal effects. Future investigations should utilize longitudinal or panel methodologies to analyze the progression of AI adoption and its organizational consequences across time.

Thirdly, the research relied on self-reported data gathered from each respondent for each survey. Despite the application of procedural remedies to mitigate common method bias, self-reported measurements may remain vulnerable to social desirability and respondent perception biases. Future research may include data from multiple sources or respondents to enhance the validity of the findings.

This research focused on organizational learning capability and digital literacy as the principal factors connecting AI adoption to organizational efficiency in Ghana’s banking industry. Future investigations may enhance the suggested framework by integrating supplementary organizational elements, including organizational culture, leadership, AI governance, or innovation capacity, and by validating the model across various industries and emerging economies to evaluate the resilience of the proposed associations within diverse institutional and technological environments.

## 9 Conclusion

This research examined the relationship between AI adoption and organizational efficiency within Ghana’s banking industry, focusing on the mediating influence of organizational learning capability and the moderating effect of digital literacy. The results indicate that the implementation of AI is associated to the improvement organizational efficiency both directly and indirectly via organizational learning capability, while digital literacy amplifies the beneficial impacts of AI adoption and organizational learning capability on organizational efficiency. These results emphasize that the organizational value of AI relies not solely on technological investment but also on supplementary organizational abilities and the digital skills of employees. By integrating these construct into a unified framework, the research enhances the Resource-Based View and provides empirical evidence from an emerging nation context. While the research concentrated on Ghana’s banking industry, the results could offer valuable perspectives for organizations in other emerging nations seeking AI-driven digital transformation. Future research should validate the proposed framework using longitudinal designs and additional organizational factors across different contexts.

## Data Availability

The original contributions presented in the study are included in the article/Supplementary material, further inquiries can be directed to the corresponding authors.
