# Supplementary material for: Artificial intelligence adoption and organizational efficiency: the mediating role of organizational learning capability and moderating role of digital literacy
Source: Front Artif Intell. 2026 Sep 30;9:1891713. doi: 10.3389/frai.2026.1891713 (PMC13661937; doi:10.3389/frai.2026.1891713)
Supplement: Supplementary file 1 [file Table_1.DOCX]

**Appendix A**: Questionnaire

**Construct and items**

**Artificial intelligence adoption (Cheng et al, 2023)**

This company has invested funds in AI technology (AIAD1)

This company has been involved in the adoption of AI technology (AIAD2)

This company has been interested in adopting AI technology to gain a competitive advantage (AIAD3)

**Digital literacy (Ng, 2012)**

I am confident in my ability to search for and evaluate information obtained from the Web for work-related purposes (COGN1)

I am familiar with issues related to web-based activities, such as cybersecurity, online search, and information use (COGN2)

ICT enables me to collaborate better with my colleagues on work projects and other work-related activities (SE1)

I frequently obtain help with my work tasks from my colleagues through the Internet and other digital communication platforms (SE2)

I know how to solve my own technical problems when using digital technologies at work (TECH1)

I can learn new digital technologies easily (TECH2)

. I keep up with important new digital technologies relevant to my work (TECH3)

I know about a wide range of digital technologies (TECH4)

. I have the technical skills I need to use ICT for my work and to create digital materials relevant to my job (TECH5)

I have good ICT skills for performing my work (TECH6)

**Organizational learning capability (Jerez-Gomez et al., 2005)**

The managers frequently involve their staff in important decision-making processes (MC1)

Employee learning is considered more of an expense than an investment. **(**MC2)

The firm's management looks favorably on carrying out changes in any area to adapt to and/or keep ahead of new environmental situations (MC3)

Employee learning capability is considered a key factor in this firm (MC4)

In this firm, innovative ideas that work are rewarded (MC5)

All employees have generalized knowledge regarding this firm’s objectives (SP1)

All parts that make up this firm (departments, sections, work teams, and individuals) are aware of how they contribute to achieving the overall objectives (SP2)

All parts that make up this firm are interconnected and work together in a coordinated fashion (SP3)

This firm promotes experimentation and innovation as a way of improving work processes (EX1)

This firm follows what other firms in the sector are doing and adopts practices and techniques it considers useful and relevant (EX2)

sources (e.g., advisors, customers, training firms) are considered useful for this firm's learning (EX3)

Part of this firm's culture is that employees can express their opinions and make suggestions regarding the procedures and methods used to carry out tasks (EX4)

Errors and failures are always discussed and analyzed in this firm at all levels (TR1)

Employees have the opportunity to discuss new ideas, programs, and activities among themselves that might be useful to the firm (TR2)

In this firm, teamwork is not the usual way of working (TR3)

The firm has instruments (e.g., manuals, databases, files, and organizational routines) that allow what has been learned from past situations to remain valid, even when employees change (TR4)

**Organizational efficiency (Atiyyah, 1999)**

Utilization of manpower (OREFF1)

Improvement in work methods (OREFF2)

Improvement in techniques and procedures over the past three years (OREFF3)

Development of employees' competencies and skills over the past three years (OREFF4)

Expansion of services achieved over the past three years (OREFF5)

Improvement in the quality of services over the past three years (OREFF6)

Speed of providing services (OREFF7)

Punctuality in providing services (OREFF8)

Responsiveness to clients' needs (OREFF9)

Utilization of available resources (OREFF10)

Cost of services (OREFF11)

**Appendix B:** Common Method Bias- Harman's single-factor test

| **Total Variance Explained** | | | | | | |
| --- | --- | --- | --- | --- | --- | --- |
| Factor | Initial Eigenvalues | | | Extraction Sums of Squared Loadings | | |
|  | Total | % of Variance | Cumulative % | Total | % of Variance | Cumulative % |
| 1 | 14.545 | 36.363 | 36.363 | 13.907 | 34.767 | 34.767 |
| 2 | 5.457 | 13.642 | 50.005 |  |  |  |
| 3 | 3.404 | 8.510 | 58.515 |  |  |  |
| 4 | 1.907 | 4.768 | 63.283 |  |  |  |
| 5 | 1.463 | 3.658 | 66.941 |  |  |  |
| 6 | 1.175 | 2.937 | 69.878 |  |  |  |
| 7 | 1.136 | 2.839 | 72.717 |  |  |  |
| 8 | 1.020 | 2.551 | 75.268 |  |  |  |
| 9 | .717 | 1.792 | 77.060 |  |  |  |
| 10 | .662 | 1.656 | 78.716 |  |  |  |
| 11 | .618 | 1.546 | 80.262 |  |  |  |
| 12 | .592 | 1.480 | 81.742 |  |  |  |
| 13 | .567 | 1.417 | 83.160 |  |  |  |
| 14 | .555 | 1.387 | 84.547 |  |  |  |
| 15 | .490 | 1.226 | 85.773 |  |  |  |
| 16 | .489 | 1.222 | 86.995 |  |  |  |
| 17 | .472 | 1.181 | 88.176 |  |  |  |
| 18 | .430 | 1.076 | 89.252 |  |  |  |
| 19 | .397 | .992 | 90.244 |  |  |  |
| 20 | .384 | .960 | 91.204 |  |  |  |
| 21 | .353 | .882 | 92.086 |  |  |  |
| 22 | .334 | .834 | 92.920 |  |  |  |
| 23 | .307 | .768 | 93.688 |  |  |  |
| 24 | .287 | .717 | 94.404 |  |  |  |
| 25 | .235 | .588 | 94.992 |  |  |  |
| 26 | .213 | .533 | 95.526 |  |  |  |
| 27 | .195 | .488 | 96.013 |  |  |  |
| 28 | .179 | .448 | 96.461 |  |  |  |
| 29 | .168 | .421 | 96.882 |  |  |  |
| 30 | .160 | .400 | 97.283 |  |  |  |
| 31 | .147 | .367 | 97.650 |  |  |  |
| 32 | .140 | .350 | 98.000 |  |  |  |
| 33 | .129 | .323 | 98.323 |  |  |  |
| 34 | .125 | .313 | 98.635 |  |  |  |
| 35 | .107 | .267 | 98.902 |  |  |  |
| 36 | .100 | .250 | 99.152 |  |  |  |
| 37 | .097 | .243 | 99.394 |  |  |  |
| 38 | .091 | .227 | 99.622 |  |  |  |
| 39 | .080 | .200 | 99.822 |  |  |  |
| 40 | .071 | .178 | 100.000 |  |  |  |
| Extraction Method: Principal Axis Factoring. | | | | | | |
